# Flexible Electronics Sensors for Tactile Multi-Touching

**DOI:** 10.3390/s9021188

**Published:** 2009-02-24

**Authors:** Wen-Yang Chang, Te-Hua Fang, Shao-Hsing Yeh, Yu-Cheng Lin

**Affiliations:** 1 Department of Engineering Science, National Cheng Kung University, Tainan 701, Taiwan; E-mail: wenyang@itri.org.tw; 2 Microsystems Technology Center, Industrial Technology Research Institute, Tainan 709, Taiwan; E-mail: FrancisYeh@itri.org.tw; 3 Institute of Mechanical and Electromechanical Engineering, National Formosa University, Yunlin 632, Taiwan; E-mail: fang.tehua@msa.hinet.net

**Keywords:** Flexible electronics, tactile, bending, organic resistance, multi-touching, printing technology, large area

## Abstract

Flexible electronics sensors for tactile applications in multi-touch sensing and large scale manufacturing were designed and fabricated. The sensors are based on polyimide substrates, with thixotropy materials used to print organic resistances and a bump on the top polyimide layer. The gap between the bottom electrode layer and the resistance layer provides a buffer distance to reduce erroneous contact during large bending. Experimental results show that the top membrane with a bump protrusion and a resistance layer had a large deflection and a quick sensitive response. The bump and resistance layer provided a concentrated von Mises stress force and inertial force on the top membrane center. When the top membrane had no bump, it had a transient response delay time and took longer to reach steady-state. For printing thick structures of flexible electronics sensors, diffusion effects and dimensional shrinkages can be improved by using a paste material with a high viscosity. Linear algorithm matrixes with Gaussian elimination and control system scanning were used for multi-touch detection. Flexible electronics sensors were printed with a resistance thickness of about 32 μm and a bump thickness of about 0.2 mm. Feasibility studies show that printing technology is appropriate for large scale manufacturing, producing sensors at a low cost.

## 1. Introduction

Multi-touch technologies are usually based on cameras and optical systems [1-4] that use emission and reflection to recognize hand gestures or objects. The use of capacitive sensing [5] is another possible sensing method, but it requires attaching a mesh-shape to achieve the interaction between transmitter and receiver. One of the most widespread multi-touch technologies uses the Frustrated Total Internal Reflection (FTIR) proposed by Jeff Han [6]. A comparison of multi-touch technologies is given in Table 1. The most common systems are expensive because they use special control hardware and require bulky equipment. A few studies, such as those done by Apple and N-trig [7,8], have focused on projected-capacitive technology for portable devices. A capacitive coupling between neighboring electrodes changes its capacitance as an object approaches the field lines projected from one electrode to another. However, projected-capacitive technology has a limited maximum size because the number of sensor electrodes needs to increase geometrically as the screen size increases. In multi-touch sensors, there is no organic resistance material between the two electrodes. It is very difficult to correctly identify which element was really touched because the mutual conduction of column and row electrodes is easily confused during the column and row scanning processes. In addition, multi-touch screens are mostly made of glass, which is difficult to bend.

Therefore, we propose a novel flexible multi-touch sensor design that prints organic thixotropic resistance materials on a top polyimide (PI) film. We also use an algorithm matrix and control system scanning to solve the array matrix for multi-touch switch identification in the tactile sensors. Flexible electronics actuators for realizing large scale and low-cost applications have been gradually developed in recent years [11,12]. Especially, flexible displays [13,14] and flexible organic transistors [15-17] have been successfully demonstrated using printing technologies. A few studies on flexible electronics sensors, usually using solid components integrated into polymer materials, have focused on small scale pressure [18], temperature [19,20], and humidity [21] sensors. Fabrication technologies for flexible electronics include screen printing [22], ink-jet printing [23,24], and the roll to roll process [25].

We have designed a gap between the bottom electrode and the resistance layer to eliminate erroneous signals during bending actions. For optimal flexible structure characteristics, a protrusion (bump) on the top membrane is used to enhance the sensing sensitivity response. A high viscosity thixotropic material is used to print the thick bump structures to reduce the diffusive effects and dimensional shrinkage after printing and curing. Mechanical properties were investigated by analysis of the deflection and stress distributions using finite element analysis (FEA). The feasibility of fabricating low-cost printable sensors for applications in flexible electronics is demonstrated.

## 2. Experimental Procedure

### 2.1. Fabrication

**Figure 1.**
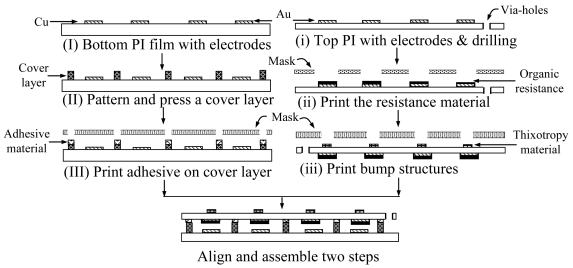
Fabrication procedures of flexible electronics sensors for large area manufacturing using screen printing technology.

This study used commercial PI films and screen printing technology to design and fabricate a flexible large area electronics sensor. PI films with copper foils are widely used in flexible electronics sensors for flexible printed circuits [26-28]. The PI film's mechanical hardness and stress characteristics are about 0.181 GPa, and 87.14 MPa, respectively [29]. The sensor structures mainly include two PI films, one cover layer, and bump protrusions. Two PI films were used as the top and bottom films of the flexible electronics sensor. The top film contains the row electrodes, the organic resistance layers, and the bump structures. The bottom film includes the column electrodes and a cover film, which was laminated into the PI film using hot pressing to form the post layer for supporting the membrane of the top film. The fabrication process mainly used a Model EKRA-E1 screen printer (Ekra, Japan), for printing materials on a flexible substrate. During the fabrication process, materials were transferred onto a substrate using a squeegee via an attached stencil mask. The fabrication process was carried out as shown in Figure 1.

First, the electrode patterns on the bottom film with a 12 μm copper foil (I), were defined for column and sensing electrodes using the photolithography method. The bottom film was then put into a solution of CuSO_4_ for electroless plating of Au, to a thickness of about 5 μm, to avoid the oxidation of the copper foil. Next, a cover layer with lattice patterns was laminated on the bottom film using hot pressing to form post structures (II). The hot-press process was performed at a temperature of 180°C for 20 minutes.

The second part of the top film, which contains the row electrodes (i), is similar to that of the bottom substrate, except that it has pass-through holes that were drilled using the punching method. Cu was coated on the side of these holes using a chemical plating to transfer the output signal from the back to the top view of top film. An organic resistance material (ii), Model EPO 4X330, with a viscosity of 8 × 10^4^ *cp* and a glass transition temperature of 190°C, was printed on sensing electrode areas using a screen stencil mask. The organic resistance was synthesized using a phenolic resin, organic solvents, filler, and carbon black. The phenolic resin is a bisphenol A (C_15_H_16_O_2_) type of organic compound with two phenol functional groups. The organic solvents included dimethylformamide (formula C_3_H_7_NO) and diethylene glycol monobutyl ether (formula CH_3_CH_2_OCH_2_CH_2_OCH_2_CH_2_OH). Dimethylformamide (*N,N*-dimethylformamide), is a hydrophilic aprotic solvent of the organic compound material that facilitates chemical reactions by polar mechanisms. Diethylene glycol monobutyl ether [2-(2-butoxyethoxy)ethanol], is the main dispersing agent for the synthetic resin and filler. The filler and carbon black are 25.6 wt% and 12.0 wt%, respectively.

For organic resistance printing, the stencil mask was a stainless steel sheet, which was made using chemical etching techniques. The printing header was a rubber squeegee of type A with a durometer of 70 and a printed angle of 45°. After top electrode and organic resistance fabrication, the film was turned over. A thixotropic material, type EPO 4X282H, with a viscosity of 10^6^ *cp* and a glass transition temperature of 150°C was printed on the top film using a screen stencil mask to form bump structures (iii). The bump structures were formed after being cured at a temperature of 150°C for 45 minutes. Finally, both PI films were aligned and assembled to form a flexible electronics sensor using adhesive (III), type Cemedine Super-X No.8008 (Cemedine Co., Japan) which was printed on the top of the post surface using screen technology.

### 2.2. Control Frame for Multi-touching

**Figure 2.**
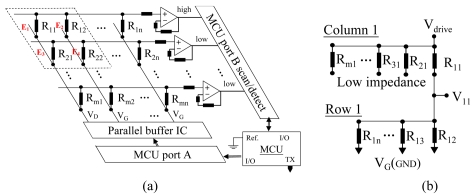
Schematic circuit of a flexible array sensor for use in multi-touch sensing applications, (a) Control system frame, and (b) the equivalent circuit of row 1 when columns inputs are 1,0, …, 0.

In general, in array switch elements for multi-touch use without a resistance layer, it is very difficult to correctly identify which elements are really touched. For example, a 2 × 2 matrix sensor with elements E_1_ to E_4_ has only an ON/OFF switch function, with R_11_, R_12_, R_21_, and R_22_ ignored, as shown in the upper left of Figure 2(a) with a dashed line. If three elements have been touched, e.g. E_1_, E_2_, and E_3_, then E_4_ will be misread as also being touched because all column and row electrodes are mutually conductive during the column and row scanning processes. An organic resistance material was printed on the surface of the sensing electrodes and an algorithm matrix and control system scanning were used to solve this problem. The schematic control system for multi-touch switching applications is shown in Figure 2(a). The system frame includes a microcontroller, a parallel buffer IC, op-amps, and an array of flexible electronics sensors. The microcontroller, model ATmega32-16MU, Atmel AVR, has three I/O ports. Port A outputs a serial high/low digital signal to the column electrode lines. Port B first sets the high or low impedance signals and then detects the sensing signals from row electrode lines. Port C transmits the sensing data. The parallel buffer IC, 74HC245, provides high driving power to avoid current decay during scanning of sensing. The non-inverting op-amp, μA741, provides a gain of about 60 to enlarge the output voltages. A flexible electronics sensor *R_mn_* (*m* × *n* array elements with a resistance of *R*) had a pitch of 5 mm with 25 × 25 elements.

In the multi-touch detection process, the parallel buffer IC outputs serial digital data to columns via port A. Only one column is the high bit, 1, and the others are the low bit, 0. Port B first sets the serial data to all rows. One row is high impedance and the others are low impedance. Then, port B is changed to detect all row data to identify which elements were touched. For instance, the serial digital data of port A, “V_D_, V_G_, …, V_G_,” outputs “1,0, …, 0” for all columns, indicating that only column 1 was driven. Then, port B sets the impedances of all rows to “high, low, …, low” and is changed to detect all row data. R_11_ can be detected using algorithm matrix operating. The equivalent circuit of the algorithm matrix for row 1 is shown in Figure 2(b). The algorithm matrix was derived using Kirchhoff's current law, as shown in equation (1). Column 1 was ignored because rows 2 to m had low impedance.


(1) $$\frac{V_{D}-V_{11}}{R_{11}}=\frac{V_{11}}{R_{12}}+\frac{V_{11}}{R_{13}}+\cdots +\frac{V_{11}}{R_{1n}}$$

Similarly, the serial digital data of port A outputs “0,1,0, …, 0” for all columns.


(2) $$\frac{V_{12}}{R_{11}}=\frac{V_{D}-V_{12}}{R_{12}}+\frac{V_{12}}{R_{13}}+\cdots +\frac{V_{12}}{R_{1n}}$$

Therefore, the algorithm matrix of row 1, *AY_1n_=0*, can be formed as follows:

(3) $$\left[\begin{array}{ccccc} \left(V_{11}-V_{D}\right) & V_{11} & \cdots & V_{11} & V_{11}\\ V_{12} & \left(V_{12}-V_{D}\right) & \cdots & V_{12} & V_{12}\\ \vdots & \vdots & \ddots & \vdots & \vdots \\ V_{1(n-1)} & V_{1(n-1)} & \cdots & \left(V_{1(n-1)}-V_{D}\right) & V_{1(n-1)}\\ V_{1n} & V_{1n} & \cdots & V_{1n} & \left(V_{1n}-V_{D}\right) \end{array}\right]\left[\begin{array}{c} Y_{11}\\ Y_{12}\\ \vdots \\ Y_{1n} \end{array}\right]=\left[\begin{array}{c} 0\\ 0\\ 0\\ 0\\ 0 \end{array}\right]$$

where *V_D_* is the driving voltage of the microcontroller, entries *V_11_* to *V_1n_* are the voltage values of row 1, and *Y_11_* to *Y_1n_* are the conductance values of the resistance at row 1, with *Y_1n_=1/R_1n_*. To solve the linear equations, Gaussian elimination was performed in row-echelon form to reduce the matrix. Then, the back-substitution method was used to solve the final equations of *Y_1n_* for multi-touch identification. The average value of resistance of elements after the voltage scanning and calculations was about 2.8 kΩ. Similarly, rows 2 to m can be scanned using the same methods to detect the signals. It is noteworthy that the microprocessor for scanning one column and solving the algorithm takes about 0.015 seconds, indicating a response frequency of 150 Hz. The sensor array of our study is 25 × 25, so this is not a problem for our system. However, we think that solving bigger matrixes will create some problems. Maybe the scanning array matrixes can be segmented and a sub-microprocessor can be used to solve the algorithm equations.

### 2.3. Mechanical Deflection

**Figure 3.**
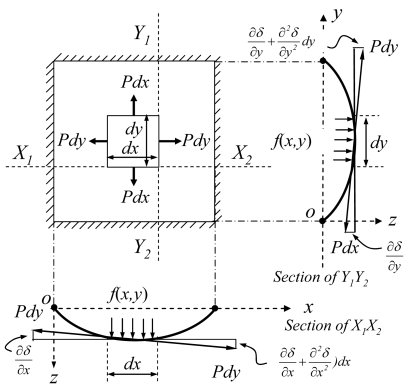
Membrane mechanical deflection of a flexible electronics sensor when a load was applied.

Consider a square plate for a linear motion equation, simply supported on all edges and subjected to a distributed load, *f*(*x, y*), in the z direction, as shown in Figure 3. Assuming the inertial force of the membrane mass is neglected and the membrane is the elastic deflection. If we consider an elemental area *dx dy*, forces of magnitude *Pdx* and *Pdy* act on the sides parallel to the *y* and *x* axes, respectively. The forces, *F*, acting along the z direction due to these forces are:

(4) $$F_{x}=P\cdot \frac{\partial^{2}\delta }{\partial x^{2}}\cdot dx\cdot dy\;\text{and}\;F_{y}=P\cdot \frac{\partial^{2}\delta }{\partial y^{2}}\cdot dx\cdot dy$$

where *P* and *δ* are the pressure and the deflection at the membrane center, respectively.

The pressure force along the z direction is *f(x,y)·dx·dy*. Hence, the membrane bending by moments distributed can be obtained for the forced transverse vibration in z direction [30,31]:

(5) $$\frac{\partial^{2}M_{x}}{\partial x^{2}}+2\frac{\partial^{2}M_{\text{xy}}}{\partial x\partial y}+\frac{\partial^{2}M_{y}}{\partial y^{2}}=P(x,y)$$

where $M_{x}=D\cdot \left[\frac{\partial^{2}\delta }{\partial x^{2}}+\upsilon \frac{\partial^{2}\delta }{\partial y^{2}}\right]$, $M_{\text{xy}}=D\cdot (1-\upsilon )\cdot \frac{\partial^{2}\delta }{\partial x\partial y}$, $M_{y}=D\cdot \left[\frac{\partial^{2}\delta }{\partial x^{2}}+\upsilon \frac{\partial^{2}\delta }{\partial y^{2}}\right]$, and $D=\frac{E\cdot {t_{m}}^{3}}{12\cdot (1-\upsilon^{2})}$.

The result of the applied pressure and the membrane deflection is given by:

(6) $$P=\frac{E\cdot {t_{m}}^{3}\cdot \delta }{(1-\upsilon )\cdot {w_{0}}^{4}}(\frac{1}{12\cdot \alpha \cdot (1+\upsilon )}+C\frac{\delta^{2}}{{t_{m}}^{2}})$$

where *t_m_* and *w_0_* are the membrane thickness and width, respectively, and *E* and *ν* are the material Young's modulus and Possion's ratio, respectively. *C* and *α* are the coefficients, which are 21.62 × (1.41-0.292 × *ν*) and 1.26 × 10^-3^ [21] respectively. The membrane characteristics are *E* = 2.5 GPa, *ν* = 0.34, *t_m_* = 50 μm, and *w_0_* = 5 mm when a pressure of *P* = 400 MPa is applied on the membrane. The central deflection is 3.59 × 10^-4^ m.

## 3. Results and Discussion

### 3.1. Finite Element Analysis

The design and simulation of a flexible electronics sensor for static tactile analysis with a large deflection were carried out using commercial FEA, ANSYS v.10.0. The effects of the membranes without bumps, with bumps, and with bumps and a resistance layer were used to analyze the deflection characteristics after a constant force was applied on the top surface. The membranes for the simulations, with a constant thickness of 50 μm, were square-shaped suspension structures. Bump sizes with width ratios of 0.2 to 1, relative to the membrane width, and with thicknesses of 0.05, 0.2, and 0.5 mm were considered. During the simulation, all elements were defined by eight nodes with three degrees of freedom at each node. A free mesh with tetrahedral-shaped elements was used to simplify the geometry creation.

**Figure 4.**
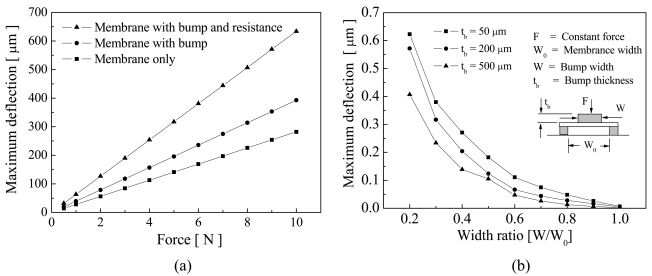
Comparison of the structural characteristics. (a) The effects of membranes without bumps, with bumps, and with bumps and a resistance layer, and (b) the effects of bump widths and thicknesses of 100, 250, and 500 μm.

For boundary condition settings, the displacements in the bottom of the posts were fixed in all degrees of freedom and the constant forces from 0.5 to 10 N were applied on the top membrane. Young's modulus, Poisson's ratio, and the density of the PI film were 2.5 GPa, 0.34, and 1.42 g/ml, respectively. The simulation results show that the maximum deflection of all cases, *δ_max_*, increased with increasing applied force, as shown in Figure 4(a), but the deflection changed less in the membranes without a resistance layer. The membrane with bumps and a resistance layer had larger deflections because the inertia force and the concentrated force increased.

The effects of width and thickness of various bumps, *W* and *t_b_*, are shown in Figure 4(b). For boundary condition settings, the displacements in the bottom of the posts were fixed and a constant force of 7.5 N was applied on top surface of the bumps. Young's modulus, Poisson's ratio, and the density of the bump material were 62 GPa, 0.3, and 1.8 g/ml, respectively. The maximum deflection curves increased significantly when the width ratios decreased, indicating that a larger bump width had a smaller deflection, which was due to the hardness of bump material being higher than that of the membrane material. In addition, membrane deflection curves decreased with increasing bump thickness. The bumps with higher thicknesses acted as rigid structures, reducing the membrane deflection. For the experimental fabrication, bumps with a width ratio of 0.4 and a thickness of 200 μm were selected in the flexible electronics sensor design.

**Figure 5.**
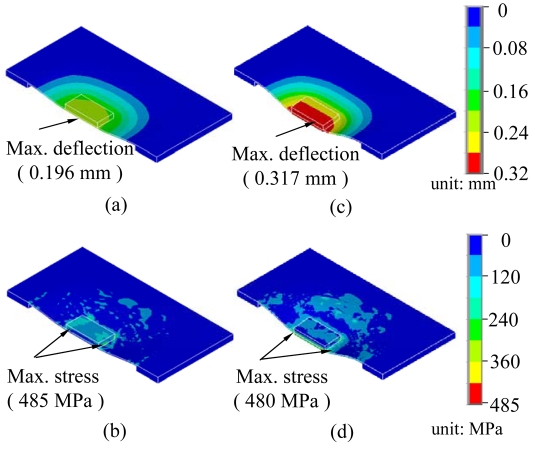
Simulation of the optimal pixel dimensions of the flexible electronics sensor at a constant force of 5 N. (a) Deflection and (b) stress distribution of a membrane with bumps, and (c) deflection and (d) stress distribution of a membrane with bumps and a resistance layer.

The mechanical characteristics analyses, deflections, and von Mises stress distributions of membranes with bumps and of those with bumps and a resistance structure, are shown in Figure 5. The dimensions of the membrane and the bumps in the simulation structures were 5 × 5 × 0.05 and 2 × 2 × 0.2 mm^3^, respectively. The maximum deflections and stresses of the membrane with a bump structure after applying a constant force of 5 N were 0.196 mm and 485 MPa, respectively, as shown in Figures 5(a) and (b). The maximum deflection was observable at the center of the membrane, but the maximum stress distributions were concentrated at the bump sides. The membrane with bumps and a resistance layer had a maximum deflection of 0.317 mm and a maximum stress of 480 MPa, as shown in Figures 5(c) and (d). The deflection of the membrane increased by 1.6 times after a resistance structure and a bump structure were added. The increased deflection was caused by an increased concentration of von Mises stress distributions and the inertia force at the bump structure. The maximum stress was less than for the membrane with bumps only because the top structures are a sandwich, which can protect the PI film.

### 3.2. Printed Sensor Characteristics

**Figure 6.**
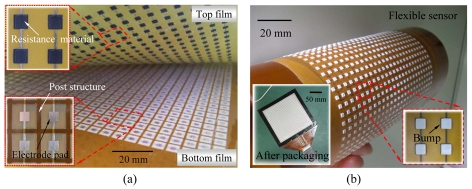
Array sensors of flexible electronics based on polyimide films for large area sensing. (a) Inside view between two PI films, including a resistance layer, posts, and electrodes, and (b) the bump structures on the top film after screen printing.

The flexible electronics of tactile sensors were successfully fabricated using two PI films. The thicknesses of the top and bottom were 50 and 125 μm. The effective area was 15 × 15 cm^2^, containing 25 × 25 arrays of sensing pads and bumps, respectively, as shown in Figure 6(a). The widths of the membrane, post, electrode lines, and sensing pads were 5, 0.8, 0.8, and 2 mm, respectively. The thickness of the sensing pad was 17 μm. The post height included a 90 μm cover and a 20 μm adhesive. Experiment results show that the organic resistance material had a uniform thickness and well-defined printing at a squeegee speed of 10 mm/s, a squeegee pressure of 70 kPa, and a separation speed of 0.4 mm/s using the single print mode. After printing, organic resistance material was cured at 190°C for 3 hours.

The bump structures, 2 × 2 × 0.2 mm^3^, were printed on the top PI film to provide a concentrated force for enhanced touch sensitivity. Results show that bump shapes had excellent morphological profiles, as shown in Figure 6(b), after being printed at a squeegee speed of 10 mm/s, a squeegee pressure of 213 kPa, and a separation speed of 0.4 mm/s using the print-print mode. Therefore, the thixotropy material with a high viscosity reduced the diffusive effects and dimensional shrinkages for printing thick structures. The sensor device after package fabrication was freely flexed without being damaged.

**Figure 7.**
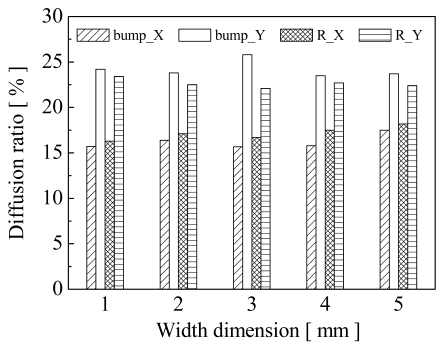
The printing diffusion ratios of the bump and resistance materials compared with original width. X and Y are parallel to and perpendicular to the printing directions, respectively.

To analyze the printed characteristics, it is necessary to measure the dimensional diffusion and average thickness. The dimensional diffusion ratio of structure dimensions after printing and curing is presented as (*W_p_ - W_i_*) / *W_i_* × *100 %*, where *W_i_* and *W_p_* are the original width and the printed width, respectively. Figure 7 shows the dimensional diffusions in the X and Y directions, which are parallel to and perpendicular to the printing directions, respectively.

Widths of 1 to 5 mm in square shapes were used to investigate the dimension effects. The results show that dimensional diffusion along the printing direction was larger than that in the Y direction. This implies that the materials were easily extruded along the printing direction during the printing process. The average percent diffusions of the resistance structures in the X and Y directions were about 24.3 % and 22.6 %, respectively. The bump material after printing had a smaller diffusion than that of the resistance material because the higher viscosity reduced the dimensional diffusive and shrinkage effects. The average percent diffusions of the bumps after printing in the X and Y directions were about 17.1 % and 16.2 %, respectively.

The values of the mean and the standard deviation for the probability density distribution of the printed resistance layer were 2.93 and 2.29 kΩ, respectively. The probability distribution at two standard deviations from mean values was 82.2 %. The range of resistance values at two standard deviations was used in the multi-touch applications in this study. The printed thicknesses were mostly consistent because the resistance value was relative to the printed thickness. Statistical computations show that the mean thickness and standard deviation of the resistance layer after screen printing were about 32.2 and 2.4 μm, respectively. The mean and standard deviation of the bump were about 192 and 10.2 μm, respectively. The thicknesses of resistance and bump layers govern the deposited volume, which can be calculated using *V = W × L × h*, where *W, L*, and *h* are the structure width, length, and thickness, respectively. The shrinkage volumes of resistance and bump layers were about 6.61 % and 3.53 %, respectively, indicating that the high viscosity had smaller shrinkages.

### 3.3. Response Analyses

The characteristic outputs of dynamic response and contact force were measured using a digital force gauge (model HF-10, ALGOL, Japan) with an accuracy of 0.1 N and a cylinder tip with a radius of 0.5 mm, a 6 axis micro-stage, an LCR meter, and a motor controller with a displacement accuracy of 1 μm.

#### 3.3.1. Bump Structure Effects

**Figure 8.**
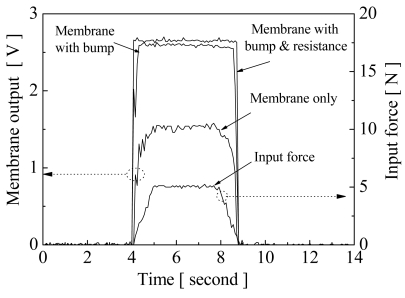
Comparison of the dynamic response effects of the membrane without bump, with bumps, and with bumps and a resistance layer.

For dynamic response measurement, a trapezoid force with a maximum force of 5 N, parallel to the y-axis in the xy-plane, was applied to the elements. The sensing mechanism of the dynamic response is based on the impedance change of resistance after contact. When a force is applied on a bump and the organic resistance layer is compressed, the sensing electronics of the top and bottom conduct the impedance changes. The equivalent circuit of sensing impedance is similar to a variable resistor. Figure 8 shows the dynamic response of membranes without bumps, with bumps, and with bumps and a resistance layer. The transient response of membrane without bumps had a delay time after the pressing force was applied. Furthermore, the response took longer to reach steady-state, at about 1.56 voltages. PI film is an elastic polymer and has an over-damping response when force is applied. The membrane with bumps reached steady-state the fastest, having no delay. The steady-state value of the membrane with bumps and a resistance layer was higher than those of the others. The bump structure and a resistance layer on the top membrane improve the response of sensing sensitivity and increase the steady-state value. During the contact process of the membrane without bumps, the post structures resisted the touch force and reduced the membrane deformation deflections when a force was applied on the top membrane. However, the membrane with a bump structure had a protrusion for enhancing membrane deflections, and it looks like an inertial mass on the center of membrane. The inertial mass can provide an inertial force to enhance the membrane dynamic response when a force is applied or released.

#### 3.3.2. Contact Force Measurement

**Figure 9.**
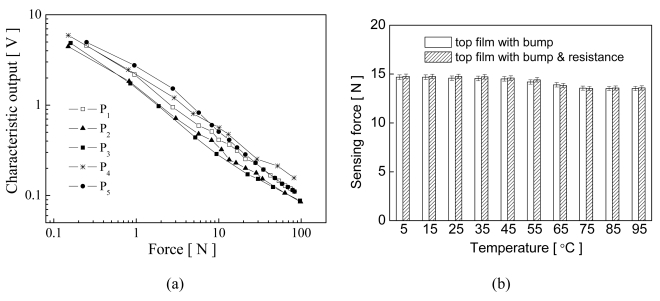
Characteristics output of flexible electronics sensors. (a) Voltage versus force at an operating frequency of 1 kHz at various pixels, and (b) steady-state output voltage response of the membrane with a bump and with bump and resistance layers at various temperatures.

For contact characteristic measurement, an external force, parallel to the y-axis in the xy-plane, was applied at the top center of the membrane. The displacements of the stages were adjusted to test the compression force versus the characteristic output at the operating frequency of 1 kHz, related to the measurement accuracy of sensing material, in ambient conditions, as shown in Figure 9(a). The symbols P_1_ to P_5_ indicate the measurement points at the center and 4 apexes of the array sensor, respectively. Results show that the impedance values sharply decreased with increasing compression force. The characteristic trend of the resistance material after fabricating was similar at the different measurement points. The characteristic output versus compression forces can approximate to linear logarithm regression. Therefore, for multi-touch switch sensing, the thresholds of the resistance response were determined according to the compression forces.

The effects of temperature variations from 5 to 95°C on different structures for switch scanning are shown in Figure 9(b). A constant force of 15 N was applied to measure the steady-state response. The responses with about 0.12 % error were transformed into sensing forces. Results show that the steady-state response slightly decreased with increasing temperature. We conclude that resistance layer became softer and had a lower resistance value at higher temperatures after a force was applied, which indicates that the resistive material has a negative temperature coefficient. However, this characteristic response can be used in multi-touch switching because the high and low signals were very clear after repeated touching at different temperatures. Although the responses at higher temperatures had small voltage changes, the multi-touch function is not influenced.

#### 3.3.3. Performance Testing

**Figure 10.**
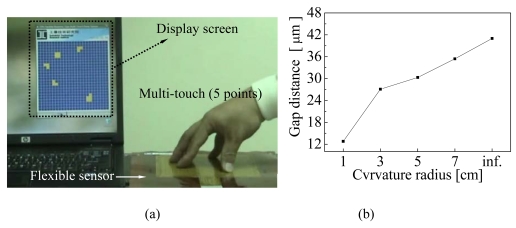
Performance testing, (a) Fingers used as input units to test multi-touch using the sensing algorithm matrix, and (b) bending testing at different curvature radii of 1, 3, 5, 7, and inf. cm.

The multi-touch testing of the flexible electronics sensors is shown in Figure 10(a). Five fingers were used to touch the flexible electronics sensor at the same time. The characteristic output of sensor was shown on a notebook screen. Blue blocks indicated untouched areas and yellow blocks indicated touched areas. The sensor can identify multi-touch operating that enables a user to interact with more than on finger at a time.

For the bending testing, flexible sensors were simply fixed on different curved surfaces of cylinder molds, with curvature radii of 3, 5, and 7 cm, to test the bending response. During experimental measurements, gap distances after differential bending were calculated using the principle of parallel capacitance, C = (ε ×A)/d. where A is the area of each plate, *ε* is the permittivity of free space, *ε* = 8.84 × 10^-12^ F/m, and *d* is the distance between the two substrates. The gap distance, at the radius of infinite curvature, is originally about 41 μm. The results of the bending tests at different curvature radii versus gap distance are shown in Figure 10(b). The gap distance at a curvature radius of 3 cm is about 27 um. However, a curvature radius of less than 1 cm easily generates erroneous signals because the small gap produces the pull-in effect. Thus, erroneous signals during the bending can be effectively eliminated when the curvature radius is over 3 cm. Moreover, the sensor was repeatedly tested using a periodic force with a digital force gauge and a motor controller. The characteristic response of the switch function was consistent after bending around a curvature radius of over 3 cm.

## 4. Conclusions

This study has successfully developed a flexible electronics sensor for multi-touch switch detection using an algorithm matrix and using screen printing technology for large-scale manufacturing. For printing thick structures, materials with higher viscosity can be used to print excellent morphological profiles, which can greatly reduce the dimension effects of diffusion and shrinkage, enhance the printing resolution, and decrease the cohesive force between the stencil mask and the sensor substrate. In addition, printed material on flexible substrates with a high surface roughness had good printed patterns and adhesive force. The gap within sensor structure provided a buffer distance to avoid erroneous contact during bending action. This sensor, with a minimum curvature radius of about 3 cm, is flexible enough to be applied anywhere without being damaged. We believe that the results of this study can be applied to many fields of flexible electronics sensors and that they provide useful information for designing and fabricating flexible large area devices.

## Figures and Tables

**Table 1.** Comparison of multi-touch characteristics.

**Item**	**ThinSight [2]**	**Jeff Han [6]**	**Apple [7]**	**Microsoft surface [9]**	**Diamond Touch [10]**	**This work**
**Sensing**	LED emitters and detectors	Infrared light and Camera	Mutual capacitance and force-sensing	Infrared and projector	Antenna and projector	Organic resistance

**Technology**	Digital I/O driving and Standard bicubic interpolation	Frustrated total internal reflection	Projected capacitive technology	Digital light processing technology	Transmitting technology	Algorithm matrix operating

**Controller**	Microcontroller and PC	Bulky equipment	Microcontroller	Bulky equipment	computer	Microcontroller

**Surface**	Acrylic	Acrylic pane	Glass substrate	Acrylic screen	Fiber glass	Polyimide

**Flexible**	Difficult	Difficult	Difficult	Difficult	Difficult	Feasible

**Sensor**	Embedding	Embedding	Embedding	Embedding	-	Screen printing

**Cost**	Medium	Medium	Medium	High	High	Low
